# P390: Hygienic risks when reusing sterile systems for infusion solution withdrawal

**DOI:** 10.1186/2047-2994-2-S1-P390

**Published:** 2013-06-20

**Authors:** FHH Brill, H Brill

**Affiliations:** 1 Dr. Brill + Partner GmbH Institute for Hygiene and Microbiology, Hamburg, Germany

## Introduction

Contamination of infusion solution can cause serious blood stream infections. In the clinical practice these single-use systems are partially reused, which may cause risks for infection [^1^].

## Objectives

A hygienic microbiological, laboratory-based study was conducted to investigate if withdrawal systems of infusion solutions can be efficiently disinfected with an alcoholic skin antiseptic. In addition, the influence of the construction of different systems was measured.

## Methods

10 of each withdrawal systems (Ecoflac® plus = System A, Kabipac® =System B, Sintetica-Bioren = System C) were contaminated with 2.5 x 10^8^ colony forming units (cfu) of the skin borne bacterium *Staphylococcus aureus* as indicator organism for contamination by skin/hand and the airborne bacterium *Kocuria rhizophila* as indicator for contamination via air. After drying of the bacteria, 9 systems were wiped with an alcoholic skin antiseptic. One system was used as growth control. At the end of the exposure time, the remaining bacterial bioburden was determined via swab sampling technique.

## Results

After disinfection of system A 7 of 9 swabs showed no growth. From one system, 1 cfu, and from another, 37 cfu were isolated. 7 swab samples from system B showed no growth; however, in 2 samples, high bacterial counts (> 300 cfu) were detected. The swab samples of the system C withdrawal system showed high bacterial counts in 6 of 9 cases (> 300 cfu), only in 2 samples, no growth was detected.

## Conclusion

Generally, it is possible to effectively disinfect withdrawal systems of infusion solution with alcoholic skin antiseptics. However, the construction of the systems influences the hygienic risk. The easier the disinfectant can be applied onto the surfaces, the more effective the procedure. Therefore, systems with smooth and easy to reach surfaces such as system A should be preferred in practical use. Nevertheless, a multiple use of the infusion systems by reprocessing with an alcoholic skin antiseptic still presents with a relatively low residual risk of infection but anyway not recommended as a microbiological contamination of the blood stream has to be avoided under all circumstances.

## Disclosure of interest

F. Brill Grant/Research support from Research was partially sponsored by B. Braun Medical AG, Switzerland., H. Brill: None declared.
